# Quantitative Classification and Prediction of Starkrimson Pear Maturity by Near-Infrared Spectroscopy

**DOI:** 10.3390/foods13233761

**Published:** 2024-11-24

**Authors:** Ruitao Lu, Linqian Qiu, Shijia Dong, Qiyang Xue, Zhaohui Lu, Rui Zhai, Zhigang Wang, Chengquan Yang, Lingfei Xu

**Affiliations:** College of Horticulture, Northwest A&F University, Taicheng Road No. 3, Yangling, Xianyang 712100, China; lrt18508779434@163.com (R.L.); lq1122ql@163.com (L.Q.); 18334754668@163.com (S.D.); jlu8218xqy@163.com (Q.X.); luzhaohui525@163.com (Z.L.); zhai.rui@nwafu.edu.cn (R.Z.); wzhg001@163.com (Z.W.); lingfxu2013@sina.com (L.X.)

**Keywords:** pear, maturity, near-infrared spectroscopy, quantitative analysis, qualitative analysis

## Abstract

Scientific evaluation of pear maturity is important for commercial reasons. Near-infrared spectroscopy is a non-destructive method that could be used for rapid assessment of pear maturity. The aim of this study was to develop a reasonable and effective method for the assessment of Starkrimson pear maturity using near-infrared technology. Partial least squares regression and five classification methods were used for analysis of the data. Among the indices used with the competitive adaptive reweighting–partial least squares regression method for quantitation, the visual ripeness index had the best modeling effect (Rp2: 0.87; root mean square error of prediction: 0.39). The classification model constructed with the visual ripeness index and post-ripeness score gave a cross-validation neural network model with the best classification effect and the highest accuracy (classification accuracy: 88.7%). The results showed that combination of quality indices with near-infrared spectroscopy was effective for rapidly evaluating the maturity of Starkrimson pears.

## 1. Introduction

The Starkrimson variety of the common pear (Pyrus communis) is an all-red and very early-ripening European pear cultivar that is grown in the United States. The fruit has fine and soft flesh, a fragrant and juicy flavor, and excellent taste, and it is much loved by consumers [1,2]. However, during the production and sales of Starkrimson pears, it is difficult for producers to judge the maturity of Starkrimson pears because a cold storage period is required after ripening [3]. Fruit that is too mature is prone to rotting and has a short shelf life. By contrast, fruit that is not mature enough has poor quality and low commercial value [4,5]. Therefore, the fruit quality and storage period must be coordinated to ensure that the fruit quality is good while also having high storage ability [6,7]. When determining the maturity of pears, the most used indicators are hardness and soluble solid content (SSC) [8,9]. However, traditional hardness and SSC measurement techniques can damage the pears and make them unsuitable for sale [7]. At the same time, the traditional means used to determine maturity are mostly sampling inspection, which struggles to accurately determine the maturity of each pear. The same batch of picked pears may have inconsistent maturity levels due to differences in growth and development conditions [10]. Non-destructive detection technology could be used to overcome these issues. Among non-destructive methods, near-infrared spectroscopy has strong penetration ability and can provide rich sample information. This method is widely used for non-destructive analysis of quality indicators such as hardness [11,12], SSC [13], titratable acidity (TA) [14], maturity [15], and internal defects in pears [16].

To date, research on the use of near-infrared spectroscopy for pear maturity has focused on predicting the hardness or SSC [17,18,19]. The maturity prediction models obtained in previous studies have high prediction accuracy and have proven that near-infrared spectroscopy is suitable for non-destructive prediction of pear maturity. However, as a commercial fruit, pears cannot fully meet the needs of consumers with a single quality indicator [20]. Moreover, during the maturation of pears, changes in color, starch content, and TA occur, in addition to changes in hardness and SSC [21]. Different quality indicators are used for pears at different levels of maturity. Therefore, multiple quality indices should be combined to provide a comprehensive description of pear maturity. Recently, maturity assessment methods using multiple quality indices have been studied in depth for apples [22,23,24,25], nectarines [26,27], mangoes [23,28], durians [29], and avocadoes [30]. The maturity indices include the Streif index, ripening index (RPI), internal quality index (IQI), visual ripeness index (VRPI), and fruit quality index (FQI). Variations in these quality indices during the ripening of fruit have been used to quantify the maturity of fruit by constructing a mathematical relationship. The use of multiple quality indicator indices provides a comprehensive representation of the fruit’s maturity and enables accurate maturity classification. Methods like this can be used to fine-tune production and help promote the sale of high-quality fruit and scientific storage and processing [31,32].

The purpose of this study was to develop a method for quantitative and qualitative analysis of the quality of Starkrimson pears using near-infrared spectroscopy. A competitive adaptive reweighting (CARS)–partial least squares regression (PLS) model was established using near-infrared spectral data and different maturity indices. This was then applied for the quantitative classification and prediction of Starkrimson pear maturity. The Starkrimson pears were assigned a maturity label according to their maturity indices and post-ripening performance. To distinguish Starkrimson pears of different maturity levels, five models were constructed using near-infrared spectral data and a linear discriminant analysis (LDA), naive Bayes (NB), k-nearest neighbors (KNN), support vector machine (SVM), or neural network (NN) algorithm. This study provides reference data for the rapid detection and quality control of Starkrimson pears, which could be used for fine-tuning production and scientific storage.

## 2. Materials and Methods

### 2.1. Samples

The experimental samples were collected from orchards in Meixian, Shaanxi Province, China (E 107°59′9″, N 34°11′40″), from 2 July 2023 (90 days after flowering, 90 DAF) to 23 July 2023 (111 days after flowering, 111 DAF). Samples were collected once a week for 4 weeks, and 100 samples were collected each time. The collected samples were stored at room temperature for 24 h and then analyzed.

### 2.2. Experimental Design

For each batch of samples, 60 samples were used for the determination of quality physicochemical indices. The remaining 40 samples were placed in cold storage at 0 °C ± 1 °C for 30 days, and then at room temperature for 3 days. Ten of these samples were selected to determine the post-ripening quality (SSC, TA, and peel color) and the edible rate [33]. The remaining 30 samples were kept at room temperature for observation, and the rot rate was calculated on day 7 [2].

*Pulp mass* = whole fruit mass − peel mass − pit mass

*Edible rate* = pulp mass/whole fruit mass × 100%

*Rot rate* = Number of bad fruit/total fruit × 100%

### 2.3. Data Collection

Visible/near-infrared spectra, color parameters (L*, a*, b*, C*, and h°), hardness, SSC, TA, and starch staining area were measured. Because the quality indices at different locations on a piece of fruit may not be consistent, each pear was divided into three sections at 120° intervals around the middle, and the sections were numbered sequentially. The results for the three sections were averaged, and the average was used as the physicochemical value for the index.

A Fourier transform near-infrared spectrometer (MPA, Bruker Optics Ltd., Etlingen, Germany) was used to collect spectral data. Before starting the measurement, the spectrometer was preheated for 40 min. The sample was connected with solid fiber. The measurement range was 4000–12,500 cm^−1^, the instrument resolution was 8 cm^−1^, and 2073 points were scanned. The data were stored in absorbance format with the internal background as the reference.

The color parameters were measured using a spectrophotometer (CR-400). The hardness was then determined using a texture analyzer (TA.XTExpress, Stable Micro Systems, Godalming, UK) with a P/2 probe. The texture analyzer was operated with a puncture speed of 1 mm/s, puncture depth of 10 mm, and trigger force of 0.049 N. SSC and TA content were determined using destructive techniques. An appropriate amount of flesh was removed from the fruit and juiced using a juicer. A 1 mL sample of the pear juice was quickly removed with a pipette and dropped into a PAL-1 digital sugar meter (ATAGO Co., Ltd., Tokyo, Japan) to determine the SSC. Another sample of pear juice (306 μL) was removed with a pipette and diluted 100 times with distilled water. A 5 mL sample of the diluted pear juice was analyzed using a pear acidity meter (GMK-835F, G-WON HITECH Co., Ltd., Seoul, Korea) to determine the TA content.

To measure the starch staining area [34], each pear was cut along the equatorial plane. A cross section of the pear was immersed in a 1% (*w*/*v*) iodine solution and 4% (*w*/*v*) potassium iodide solution for 1 min, and then dried in air for 5 min. A photograph of the starch staining area was taken with a mobile phone.

### 2.4. Indices for Assessing Maturity

The main fruit maturity evaluation indices are the Streif Index [22], RPI [28], IQI [26], and VRPI [25]. These indices were calculated using the following equations:

$$\mathrm{Streif}\;\mathrm{Index}=\frac{F}{SSC\times SPI}$$

$$\mathrm{RPI}=\ln\frac{100\times F\times TA}{SSC},\mathrm{and}$$

$$\mathrm{IQI}=\ln\frac{100\times F\times TA\times L\times h}{SSC*C}$$

where *F* is pulp hardness, *TA* is titratable acidity, *SPI* is the starch staining index, SSC is the soluble solid content, L* is the brightness, C* is the chroma saturation, and *h°* is the hue angle.

$$\mathrm{VRPI}=e^{\frac{1}{2}\times \sin\alpha \times (\sum_{n=1}^{m}\left(Pn\times Pn+1+P1\times Pm\right)}$$

where α is the angle surrounded by two adjacent variables, *P_n_* is the value of the *n*th variable, and *P_m_* is the total number of variables.

The VRPI is calculated using the geometric area of the radar map [25]. First, parameters with low correlations in each maturity index were selected, and then multiple maturity quality parameters for the same sample were integrated into the same central point. The numerical axis radiates outward from the center of the circle, and the geometric area of the radar map is calculated and used to obtain the VRPI.

### 2.5. Data Processing and Modeling

#### 2.5.1. Outlier Removal and Spectral Preprocessing

During analysis of the fruit, two samples were found to have internal lesions. The data from these fruit were deleted as outliers. In the subsequent modeling analysis, the total number of samples was 238.

Spectral preprocessing was performed to establish the analytical model. Spectral signals are susceptible to interference from stray light and noise, which greatly affects the accuracy of the model. Therefore, it is necessary to preprocess the spectrum. Common spectral preprocessing methods include smoothing, derivative, standard normal variable transformation (SNV), and multivariate scattering correction (MSC) [35]. In this study, five methods were selected to preprocess the spectrum: Savitzky–Golay smoothing (SG), standard normal variable transformation, multivariate scattering correction, SNV plus first derivative, and MSC plus second derivative.

#### 2.5.2. Spectral Dimensionality Reduction

High-dimensional spectral data carry a large amount of information, which both increases the computational workload in the modeling process and introduces too much irrelevant information that decreases the model’s accuracy [36]. Reductions in the dimensionality of spectral data can reduce the calculation workload and improve the model’s accuracy. In this study, the CARS method was used to reduce the dimensionality of the spectral data. CARS is an algorithm that is used to select spectral wavelength points according to the regression coefficient [37]. The importance of wavelength points is determined by comparing the absolute values of regression coefficients. During the operation of the algorithm, important wavelengths are selected according to adaptive reweighting sampling. Then, the optimum wavelength combination is determined from the smallest subset of the root mean square error (RMSE) in the cross-validation process.

#### 2.5.3. Quantitative and Classification Model of Starkrimson Pear Maturity

Before establishing the quantitative model, the samples were divided into a training set (n = 180) and a test set (n = 58) in a 3:1 ratio using the Kennard–Stone algorithm. The preprocessed spectral data were used to construct quantitative models of different maturity indices using the PLS method. The parameters used to evaluate the performance of the quantitative models were the root mean square error of cross-validation (RMSECV), the root mean square error of prediction (RMSEP), Rcv2, and Rp2. The quantitative model with the lowest RMSECV and RMSEP and the closest Rcv2 and Rp2 to one was selected as the best.

Classification models were developed using data from the best quantitative model. The classification models were constructed using Classification Learner in MATLAB (2023b) and an LDA (discrimination type: linear; optimizer: Bayesian Optimization), NB (distribution name: kernel; kernel type: Gaussian; standardized data: true), KNN (number of neighbors: 3; distance metric: Minkowski (cubic); distance weight: inverse distance squared; standardized data: true), SVM (kernel function: linear; box constraint level: 0.5901; multiclass encoding: one-to-one; standardized data: true), or NN (number of neurons in hidden layers: 50; maximum number of iterations: 200; activation function: Sigmoid; regularization strength (lambda): 0.00030942; standardized data: true) algorithm. LDA is a classical linear supervised classification method that is widely used for classification recognition [38]. NB is one of the most extensive classification models. The algorithm is based on Bayes’ theorem and the independent assumption of feature conditions [39]. KNN is an efficient and simple nonparametric classification method [40]. SVM is a supervised learning classification method that is based on statistical learning theory [41]. NN can be used for nonlinear adaptive information processing, and it performs well for data processing and analysis [42]. These five classification algorithms have been widely used in research for fruit classification [43]. The samples were divided into ripeness categories according to the fruit scores. The classification models’ performance was evaluated using the classification accuracy (CA) and confusion matrix. The best classification model was selected according to the highest classification accuracy and the best performance of the confusion matrix.

## 3. Results and Discussion

### 3.1. Maturity Analysis

#### 3.1.1. VRPI

**Figure 1:**
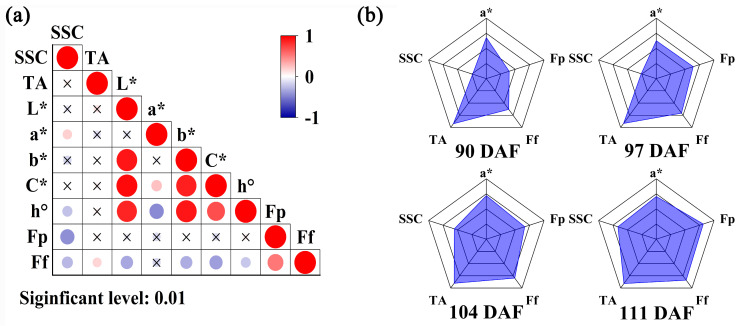
VRPI calculation process diagram. (**a**) Correlation analysis heatmap of quality indicators. (**b**) Average radar chart of samples from four sampling periods.

Calculation of the VRPI requires correlation analysis of each quality index. To describe the maturity of Starkrimson pears comprehensively, quality indices that could represent the fruit state and had low correlations were selected. Among the nine quality indices measured, SSC and TA represented the flavor of the fruit, L*, a*, b*, C*, and h° represented the color of the fruit, and fruit peel hardness (Fp) and flesh firmness (Ff) represented the taste of the fruit. Pearson correlation analysis was conducted for nine quality indices using Origin (2022) software, and the significance level was 0.01 (Figure 1a). The correlation between TA and Ff was 0.18, while TA was not correlated with the other seven indices. a* was not correlated with L*, b*, Fp, or Ff, and had small correlations with C* and h°. During the ripening process of Starkrimson pears, the color of the peel changes from dark red to bright red. a* mainly describes the red and green colors of the fruit. According to these results, SSC, TA, a*, Fp, and Ff were selected as the calculation parameters for the VRPI index. First, all parameters were normalized, and then parameters other than SSC and a* were reverse-processed according to the change trend in the maturation process. Finally, the VRPI of each pear was calculated using the described formula. Figure 1b shows the average radar map of the four batches of ‘Starkrimson’ pears. The radar map clearly shows that as the maturity increased, the VRPI gradually increased.

#### 3.1.2. Statistics of Each Maturity Index

Table 1 shows the statistical results for the maturity indices of the four batches. Each maturity index showed a relatively stable trend of change. Except for the VRPI, all maturity indices showed a downward trend. The changes in the maturity indices between adjacent batches of pears were small. These results show that the ripening of Starkrimson pears is a continuous and relatively stable process for each maturity index, and that there are no sudden or large content changes. Therefore, there is a strong basis for evaluating the maturity of Starkrimson pears using multiple quality indices. This also establishes a solid foundation for quantitation of Starkrimson pear maturity and shows that it is feasible to analyze and characterize the maturity of Starkrimson pears using maturity indices.

### 3.2. Spectral Analysis

**Figure 2:**
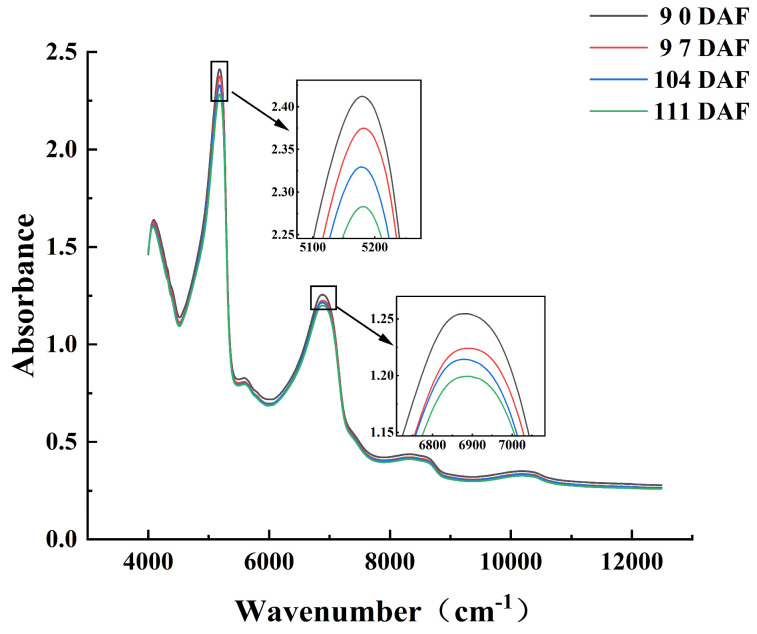
Average spectra of Starkrimson pears collected during four different periods.

Near-infrared spectra are generated by frequency doubling and co-frequency absorption of the fundamental frequency vibrations of the material molecules, which changes the energy level and generates the absorption spectrum. The absorption spectrum (also known as the vibration spectrum) appears in the form of a spectral band. Overlap of bands frequently occurs in the near-infrared region. Near-infrared spectra contain rich material structure and chemical composition information, and the near-infrared absorptions of different groups show obvious differences [44]. Because the absorption bands of hydrogen-containing groups (C-H, O-H, and N-H) can often be observed in the near-infrared region, near-infrared spectroscopy can be applied for the detection of the physical and chemical parameters of hydrogen-containing organic compounds (e.g., fruit and vegetables). The average spectra of the four batches of Starkrimson pears (Figure 2) showed that the absorbance decreased as the harvest date increased. The average spectrum of each batch showed five main absorption peaks. A strong absorption peak near 5150 cm^−1^ was attributed to the antisymmetric stretching and bending vibrations of water molecules. Peaks at 5555–5882 cm^−1^ and 8264–8696 cm^−1^ were assigned to the first and second frequency-doubling regions of the C-H stretching vibration, respectively. The peaks near 5620 cm^−1^ and 8310 cm^−1^ were attributed to the combined spectral bands of liquid water. Finally, peaks near 6900 cm^−1^ and 10,300 cm^−1^ were assigned the first and second frequency-doubling regions of the O-H stretching vibration, respectively [45]. These absorption peaks mainly contain the spectral information of water, carbohydrates, and carboxylic acid in Starkrimson pears. Differences in the spectra of the different batches show that the near-infrared spectral information can be used to effectively characterize changes in pears during the ripening process.

### 3.3. Quantitative Analysis

CARS-PLS models were constructed using different preprocessing methods with the spectral data and maturity indices. The verification method was five-fold cross-validation. The relevant model parameters are shown in Table 2. All maturity indices showed high predictability under the SNV + first derivative pretreatment. Compared with other maturity indices, the VRPI gave the best model prediction performance with all preprocessing methods. Considering the overall value of the performance parameters of each model, the VRPI model with SNV + first derivative preprocessing was selected as the best model. The performance parameters of this model were Rcv2 = 0.90, RMSECV = 0.17, Rp2 = 0.87, and RMSEP = 0.39. The VRPI model with MSC + second derivative preprocessing also had high Rcv2 and Rp2 values, but they were slightly worse than those of the VRPI model with SNV + first derivative preprocessing. For the remaining maturity indices, the model built using the IQI was better than the other models. The Rcv2 and Rp2 for this model were above 0.70. Compared with the models constructed using the VRPI and IQI, the model constructed using the RPI had lower accuracy. This may be because of the lack of fruit color parameters in the calculation process of the RPI, which means that it will fail to describe the ripeness of fruit comprehensively. However, the Streif index performed poorly in all models, which may be because of the poor rating results of the starch staining grade or the inapplicability of starch staining for the characterization of Starkrimson pear maturity, resulting in a low correlation between the Streif index and the spectrum.

**Figure 3:**
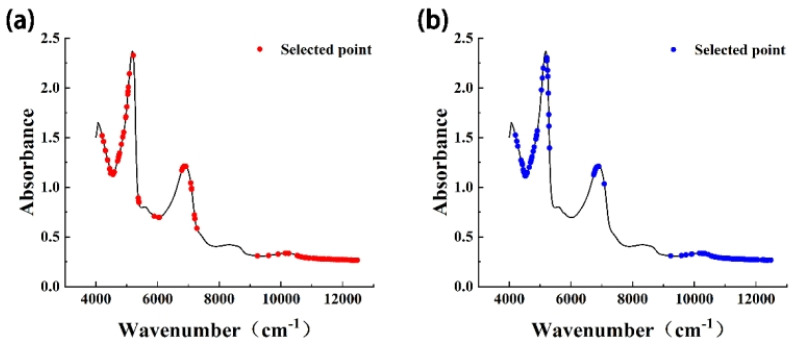
Schematic diagram of characteristic wavenumbers. (**a**) SNV + first derivative. (**b**) MSC + second derivative.

In the process of model construction, application of the CARS method reduced the original input spectral points from 2203 to 30–194 (Table 2). Dimensionality reduction of spectral data can improve the calculation rate of the model, reduce the proportion of redundant spectral information, and improve the prediction effect of the model. The wavenumber points selected by CARS were mainly concentrated in the regions of 4000–5000 cm^−1^ and 10,300–12,500 cm^−1^, and a small number were distributed in the region of 6800–7000 cm^−1^ (Figure 3). The absorptions in these regions are mainly the vibration absorptions of C-H and O-H, and the corresponding compounds are mainly natural organic compounds such as sucrose, starch, cellulose, and lignin. In the growth and development process of Starkrimson pears, changes in the contents of these compounds will be reflected in the quality indices (e.g., hardness, SSC, and TA). Therefore, the producer could simply determine pear maturity using the value of the quality index. In the near-infrared spectrum, differences in the contents of these compounds were observed as changes in the magnitude of the absorbance, and this provides a basis for the use of near-infrared spectral data to determine the maturity of Starkrimson pears.

**Figure 4:**
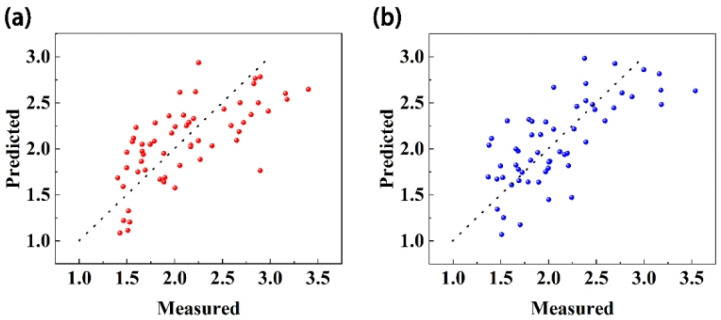
VRPI prediction effect diagram. (**a**) SNV + first derivative. (**b**) MSC + second derivative.

The quantitative model results for each maturity index showed that reasonable construction of the relationship of each quality index and selection of appropriate spectral preprocessing methods could improve the correlation between the spectrum and maturity, and this could be used to build a better quantitative model. Calculation of the VRPI considers the change trend for each quality index in the ripening process and provides a good representation of the maturity of Starkrimson pears. Using the five preprocessing methods, the model constructed with the VRPI had the best performance. Therefore, the VRPI was the best maturity indicator. Among the five preprocessing methods, SNV + first derivative and MSC + second derivative performed better than the other three. Figure 4. demonstrates the prediction results of VRPI under these two preprocessing methods.

### 3.4. Qualitative Analysis

#### 3.4.1. Maturity

Because Starkrimson pears need to be refrigerated before they can be eaten, it is necessary to include post-ripening when characterizing the pears according to ripeness [3]. After considering the marketability and shelf life of Starkrimson pears, a*, SSC, TA, the edible rate, and the rot rate were selected for evaluation after ripening. These five quality indices were normalized. Among them, a*, SSC, and the edible rate were treated in the forward direction, while TA and the rot rate were treated in the reverse direction. After normalization, the five quality parameters were assigned the same mass (0.2), and the sum of the mass for each batch was calculated as the post-ripening score of the batch (Table 3). After the VRPI was normalized, the final fruit score (Fs) was obtained by adding the post-ripening score and VRPI of the same batch. According to the fruit score, the pears were classed as unripe if 0.8 < Fs, ripe if 0.8 ≤ Fs < 1.2, and over-ripe if 1.2 ≤ Fs. The 238 Starkrimson pears were divided as follows: 120 unripe, 67 ripe, and 51 over-ripe.

#### 3.4.2. Classification of Starkrimson Pears with Differences in Maturity

In the quantitative prediction model using the VRPI, the characteristic wavelengths under the two optimum preprocessing methods (SNV+ first derivative and MSC + second derivative) were taken as the input variable. From the results of the maturity classification (Section 3.4.1), a maturity classification model for Starkrimson pears was constructed. There were two validation methods: five-fold cross-validation and independent test set validation (179 samples for calibration and 59 samples for validation).

The classification effects of the 10 classification models with the SNV + first derivative method are shown in Table 4. In the cross-validation model, the classification accuracy of each model was above 80%. Among them, the NN model gave the best classification model, with a classification accuracy of 88.7%. The number of true positives with this model was high (over-ripe: 36/51; ripe: 54/67; unripe: 120/120). The next most accurate model was the SVM model, with an accuracy rate of 88.2%. Among the test set validation models, the naive Bayes model had the worst performance, with a classification accuracy of only 79.7%. The best validation model was the NN model, with a classification accuracy of 91.5% and the highest number of true positives (over-ripe: 10/12; ripe: 13/16; unripe: 31/31).

Table 5 shows the classification effects of 10 classification models using the MSC + second derivative method. Except for the two naive Bayes models and the LDA model verified by the test set, all models had a classification accuracy of >80%. Using the two verification methods, the best classification model was the SVM model. The classification accuracy of the cross-validated SVM model was 87.4%, and the number of true positives was as follows: over-ripe 36/51, ripe 52/67, and unripe 120/120. The classification accuracy of the SVM model verified by the test set was higher (89.8%) and the number of true positives was as follows: over-ripe 10/13, ripe 13/16, and unripe 30/30.

According to the true positive results for the various classification models, the classification accuracy of each model for unripe fruit reached 100%. However, there were some cases of misclassification between ripe and over-ripe fruit, which are easy to confuse. The reason for this may be the high similarity in the spectra of over-ripe and ripe fruit. Alternatively, the Fs was not suitable for distinguishing between over-ripe and ripe fruit and needs to be improved. The best algorithms in each model were SVM and NN, which give similar results for both types of preprocessing. At the same time, the performance of the model treated with SNV + first derivative was slightly better than that of the model treated with MSC + second derivative. The construction of the classification model should ensure high accuracy and pay attention to the number of true positive samples in the model so that it meets practical application requirements. The classification model constructed by the SVM and NN algorithms can identify unripe fruit perfectly and distinguish ripe fruit from over-ripe fruit. After further optimization, this model could be applied to maturity classification of Starkrimson pears in actual production.

## 4. Conclusions

Near-infrared spectroscopy was combined with maturity indices to construct quantitative and qualitative prediction models for Starkrimson pear maturity. This gave a feasible method for quantification of the maturity of Starkrimson pears. Among the maturity indices, the VRPI gave the best description of the maturity of Starkrimson pears, and the best model Rp2 in quantitative prediction was 0.87. Through qualitative analysis of the VRPI, the best cross-verified classification model CA was 88.7%, which could identify and classify unripe, over-ripe, and ripe fruits well. Unripe fruit was accurately identified on all occasions, but there were some instances of misclassification with ripe and over-ripe fruit. A fruit score was proposed for the post-ripening of Starkrimson pears. The fruit score can describe the ripening state of Starkrimson pears and be used to determine their commodity value. This method could be used for maturity classification during the production and sale of Starkrimson pears.

## Figures and Tables

**Table 1:** Statistical results for maturity indices of four batches of pears.

Time of Sampling	Maturity Index			
	Streif	RPI	IQI	VRPI
90 DAF	0.57 ± 0.17	2.28 ± 0.27	8.37 ± 0.16	1.59 ± 0.19
97 DAF	0.55 ± 0.19	2.22 ± 0.19	8.30 ± 0.23	1.81 ± 0.24
104 DAF	0.42 ± 0.11	2.02 ± 0.20	8.08 ± 0.18	2.27 ± 0.37
111 DAF	0.41 ± 0.13	1.94 ± 0.19	8.02 ± 0.25	2.63 ± 0.55

**Table 2:** Statistics of quantitative prediction results of Starkrimson pear maturity.

Ripeness Index	Pretreatment	PLS Factors	Variables	Training Set		Prediction Set	
				R_cv_^2^	RMSECV	R_p_^2^	RMSEP
Streif	SG	12	46	0.59	0.11	0.58	0.12
RPI		11	40	0.66	0.15	0.63	0.19
IQI		9	168	0.75	0.13	0.73	0.15
VRPI		11	71	0.76	0.27	0.74	0.31
Streif	MSC	10	40	0.61	0.11	0.60	0.15
RPI		11	54	0.71	0.14	0.69	0.19
IQI		9	46	0.77	0.12	0.77	0.15
VRPI		12	82	0.80	0.24	0.79	0.30
Streif	MSC + 2nd D	13	194	0.72	0.09	0.68	0.20
RPI		11	168	0.71	0.14	0.70	0.21
IQI		11	109	0.78	0.12	0.77	0.23
VRPI		14	146	0.86	0.20	0.86	0.43
Streif	SNV	8	30	0.55	0.12	0.54	0.14
RPI		10	54	0.69	0.14	0.66	0.19
IQI		8	109	0.76	0.12	0.75	0.15
VRPI		11	62	0.78	0.25	0.76	0.30
Streif	SNV + 1st D	9	109	0.64	0.10	0.62	0.14
RPI		14	126	0.80	0.11	0.76	0.18
IQI		9	54	0.81	0.11	0.80	0.15
VRPI		15	126	0.90	0.17	0.87	0.39

**Table 3:** Statistics of post-ripening scores of Starkrimson pears collected during four sampling periods.

Date	a*	SSC	TA	Edible Rate	Rot Rate	Post-Ripeness Score
90 DAF	0.00	0.00	0.00	0.00	0.37	0.07
97 DAF	0.38	0.76	0.00	0.71	0.33	0.44
104 DAF	0.60	0.62	1.00	1.00	1.00	0.84
111 DAF	1.00	1.00	1.00	0.71	0.00	0.74

**Table 4:** Statistical results of classification model SNV + first derivative.

Validation	Model	Confusion Matrix					Classification Accuracy (%)
Cross-validation	SVM		Predicted				88.2
		Actual		O	R	U	
			O	36	15	0	
			R	13	54	0	
			U	0	0	120	
	KNN		Predicted				80.7
		Actual		O	R	U	
			O	28	23	0	
			R	23	44	0	
			U	0	0	120	
	NN		Predicted				88.7
		Actual		O	R	U	
			O	39	12	0	
			R	15	52	0	
			U	0	0	120	
	NB		Predicted				80.7
		Actual		O	R	U	
			O	32	19	0	
			R	26	41	0	
			U	0	1	119	
	LDA		Predicted				84.0
		Actual		O	R	U	
			O	35	16	0	
			R	20	46	1	
			U	0	1	119	
Test set validation	SVM		Predicted				84.7
		Actual		O	R	U	
			O	9	3	0	
			R	6	10	0	
			U	0	0	31	
	KNN		Predicted				84.7
		Actual		O	R	U	
			O	9	3	0	
			R	6	10	0	
			U	0	0	31	
	NN		Predicted				91.5
		Actual		O	R	U	
			O	10	2	0	
			R	3	13	0	
			U	0	0	31	
	NB		Predicted				79.7
		Actual		O	R	U	
			O	7	5	0	
			R	7	9	0	
			U	0	0	31	
	LDA		Predicted				81.4
		Actual		O	R	U	
			O	6	6	0	
			R	4	12	0	
			U	0	1	30	

**Table 5:** Classification results of MSC + second derivative.

Validation	Model	Confusion Matrix					Classification Accuracy (%)
Cross-validation	SVM		Predicted				87.4
		Actual		O	R	U	
			O	36	15	0	
			R	15	52	0	
			U	0	0	120	
	KNN		Predicted				83.2
		Actual		O	R	U	
			O	32	19	0	
			R	21	46	0	
			U	0	0	120	
	NN		Predicted				85.3
		Actual		O	R	U	
			O	34	17	0	
			R	16	51	0	
			U	1	1	118	
	NB		Predicted				79.4
		Actual		O	R	U	
			O	24	27	0	
			R	22	45	0	
			U	0	0	120	
	LDA		Predicted				81.1
		Actual		O	R	U	
			O	31	20	0	
			R	21	45	1	
			U	1	2	117	
Test set validation	SVM		Predicted				89.8
		Actual		O	R	U	
			O	10	3	0	
			R	3	13	0	
			U	0	0	30	
	KNN		Predicted				86.4
		Actual		O	R	U	
			O	9	4	0	
			R	4	12	0	
			U	0	0	30	
	NN		Predicted				84.7
		Actual		O	R	U	
			O	8	5	0	
			R	4	12	0	
			U	0	0	30	
	NB		Predicted				78.0
		Actual		O	R	U	
			O	5	7	1	
			R	3	11	2	
			U	0	0	30	
	LDA		Predicted				76.3
		Actual		O	R	U	
			O	4	9	0	
			R	5	11	0	
			U	0	0	30	

## Data Availability

The original contributions presented in this study are included in the article; further inquiries can be directed to the corresponding author.
